# Development of a new ‘ultrametric’ method for assessing spawning progression in female teleost serial spawners

**DOI:** 10.1038/s41598-020-66601-w

**Published:** 2020-06-15

**Authors:** Kelli C. Anderson, Maud Alix, Katerina Charitonidou, Anders Thorsen, Grethe Thorsheim, Kostas Ganias, Thassya C. dos Santos Schmidt, Olav Sigurd Kjesbu

**Affiliations:** 1 Institute of Marine Research, PO Box 1870 Nordnes, NO-5817 Bergen, Norway; 2 Present Address: Institute for Marine and Antarctic Studies, University of Tasmania Newnham Campus, Private Bag 1370, Newnham, Tas, 7248 Australia; 3 Department of Biology, Aristotle University of Thessaloniki, 54636 Thessaloniki, Greece

**Keywords:** Marine biology, Population dynamics

## Abstract

The collection and presentation of accurate reproductive data from wild fish has historically been somewhat problematic, especially for serially spawning species. Therefore, the aim of the current study was to develop a novel method of assessing female spawning status that is robust to variation in oocyte dynamics between specimens. Atlantic cod (Barents Sea stock) were used to develop the new ‘ultrametric’ method, that is based on the progressive depletion of the vitellogenic oocyte pool relative to the rather constant previtellogenic oocyte (PVO) pool. Fish were subsequently partitioned into one of four categories that accurately reflected changes in their oocyte size frequency distribution characteristics and gonadosomatic index throughout spawning. The ultrametric method overcomes difficulties associated with presence of bimodal oocyte distributions, oocyte tails, lack of clear hiatus region, and presence of free ova, and can be implemented at a single sampling point. Much of the workflow is fully automated, and the technique may circumvent the need for histological analysis depending on the desired outcome. The ultrametric method differs from the traditional autodiametric method in that PVOs can be separated by ultrasonication and then enumerated, and ovarian homogeneity is not a mandatory requirement per se. The method is designed for determinate spawners but might be extended to include indeterminate spawners.

## Introduction

The collection and integration of reproductive data is essential for effective fisheries advice^1^ as information relating to age and size at sexual maturity and reproductive capacity inform stock status, recruitment potential, and biomass estimates^2,3^. While significant efforts have been made by marine laboratories^4,5^ as well as by advisory bodies (e.g. www.ices.dk) to standardise reproductive data generation and improve the overall quality, there remain several instances where the application of current methods involves major uncertainty, e.g. macroscopic maturity staging, asking for additional histology^6,7^. There are also challenges related to the corresponding microscopic assessment^8^. A particularly key issue is the type of oocyte recruitment pattern in question, which is not only relevant in fecundity studies but also for understanding spawning performance of the species^9^. For example, Harðardóttir and colleagues^10^ monitored the increase in gap (‘hiatus’) size that occurs between previtellogenic oocytes (PVOs) and vitellogenic oocytes (VOs) as ovaries of *Gadus morhua* advanced. As such, this ‘hiatus limit method’ relies on the standard assertion that this gadoid is a strictly determinate spawner with no oocytes between the major PVO and VO cohorts, which facilitates non-subjective placement of the hiatus boundaries. However, we encountered difficulties when using this technique with *G. morhua* and found that the hiatus limits were not always clear (this study). Thus, the required stringency of fecundity style may certainly be problematic^11^ when studying oocyte size frequency distributions (OSFDs).

In contrast to the hiatus limit method, the autodiametric method^12^ utilises changes in the mean size, SD and skewness of VO OSFDs to track individual reproductive development over time. However, the main purpose of this frequently used, fully automatic method, is to estimate individual fecundity prior to spawning by relating mean VO size with the corresponding oocyte packing density (OPD)^13^. The proper establishment of this calibration curve requires ovarian homogeneity, which is typically seen in pre-spawners but not in spawners, where ovulated oocytes congregate in the ovarian lumen before egg release (e.g. sole, *Solea solea*^14^). So, despite the notation that the autodiametric method also works on species showing complex OSFDs, such as VO bimodality in pre-spawning *Reinhardtius hipploglossoides*^13^, it is not intended for examination of spawning ovaries. Alternatively, the historic ‘stage of spawning method’ is designed for use during spawning and uses VO SD as criterion^15^ but encounters similar problems to the above-mentioned hiatus limit method. Again, to be applied, it is essential to properly define the purpose of the examination, confirm whether the underlying principle of oocyte development dynamics remain consistent among individuals, and select the right method. Here, we identify a need to design a modern method that will facilitate ovulatory cycle studies, i.e. when addressing oocyte growth and recruitment processes happening within a relatively short time frame between successive egg batches^15,16^, to improve the resulting accuracy and precision in every step of the analytic protocol.

Unlike experimental or aquaculture studies, reproduction assessment methods in the field must be compatible with on-going stock survey programs or sequential processing of landings that often happen within a few days. Logically, this utilisation of discreet sampling complicates tracking of gonad dynamics, especially during the intense, often relative short spawning season. Therefore, the aim of the current study was to develop a novel method of assessing spawning status that is robust to variation in oocyte dynamics between individuals and can be used at discrete sampling points during the spawning season. To do so, we used wild *G. morhua*, a serial spawner which is traditionally considered a determinate species as the model. We combined advanced image analysis techniques with histology to get an in-depth understanding of oocyte development patterns, not only regarding VOs but also for the much smaller PVOs, and used this data to develop a ranking system that relies on progressive depletion of the VO pool throughout spawning. Collectively, this ranking system is named the ultrametric method where ‘metric’ refers to automated whole-mount measurements as for the autodiametric method, and ‘ultra’ reflects the incorporation of ultra-small oocytes (PVOs) into the calculation, and the use of ultrasonication to separate the oocytes prior to their staining and measurement. Using the new ultrametric method, we partitioned females into one of four categories, beginning with pre- and early-spawning fish (category 1), through to very late- and post-spawning fish (category 4). We addressed three main hypotheses as a means of validating the ultrametric method: (1) Post-ovulatory follicles (POFs) would accumulate throughout spawning due to persistence of POFs in cold-temperate environments^17^; (2) As cod is a capital breeder^18^, body condition index should decline throughout spawning, and (3) The gonadosomatic index (GSI) is expected to drop markedly during the course of spawning^19^.

## Results

### Assessment of oocyte size frequency distributions

**Figure 1:**
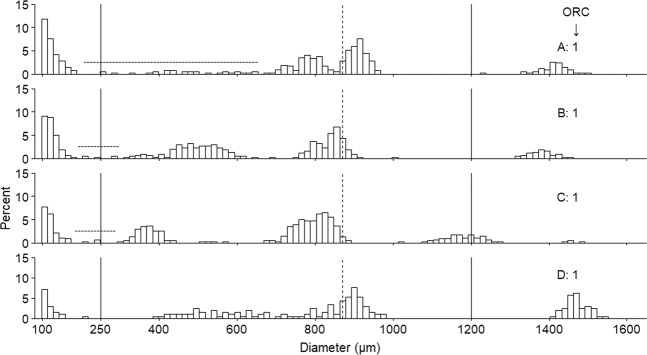
Examples of assumingly atypical oocyte frequency distribution (OSFD) of spawning individuals. (**A–C**) OSFDs demonstrating the subjective nature of hiatus placement. Placement of the gap limits should theoretically be placed at the upper and lower edge of the PVO and VO cohort, respectively. However, the limits could subjectively be placed at multiple positions within the region indicated by the horizontal dashed line. Furthermore, panel D exemplifies an OSFD where the VO cohort has an unusually long ‘tail’. The upper and lower thresholds used for calculation of oocyte ratio (OR) are shown by the solid vertical lines, the vertical dashed line is the approximate point at which oocytes enter final oocyte maturation (FOM)^20^. All fish in this figure have an oocyte ratio category (ORC) of 1 for standardisation purposes. Oocytes <250 µm are PVOs, 250 to 1200 µm are developing oocytes (VO and FOM), and >1200 µm hydrated or ovulated oocytes. Panel C exemplifies an individual with ‘swelling’ oocytes which are at the end of FOM (see mode at ≈1200 µm).

While measures such as PVO-VO hiatus size have historically been used as an approach to assess progression through the spawning period for *G. morhua*^10^, this hiatus limit method was currently found unsuitable for ~15% of individuals as placement of the gap limits was too subjective (examples in Fig. 1A–C).

**Figure 2:**
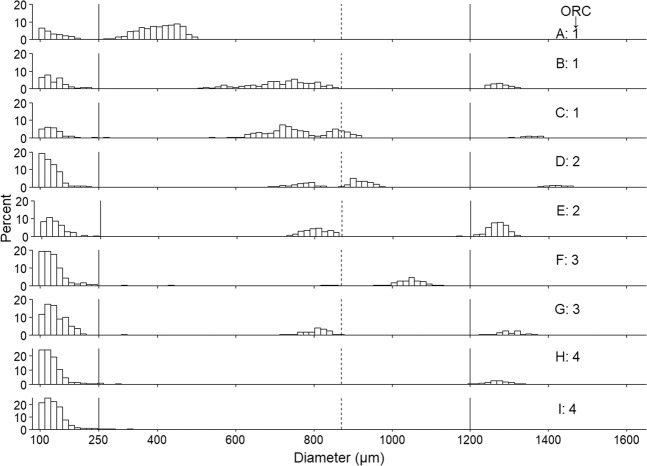
Representative OSFDs belonging to each of the four ORCs. OSFDs from pre-spawning (**A**), early-spawning (**B,C**), mid-spawning (**D,E**), late-spawning (**F,G**), very late-spawning (**H**), and post-spawning fish **(I**). Other details as for Fig. 1.

**Figure 3:**
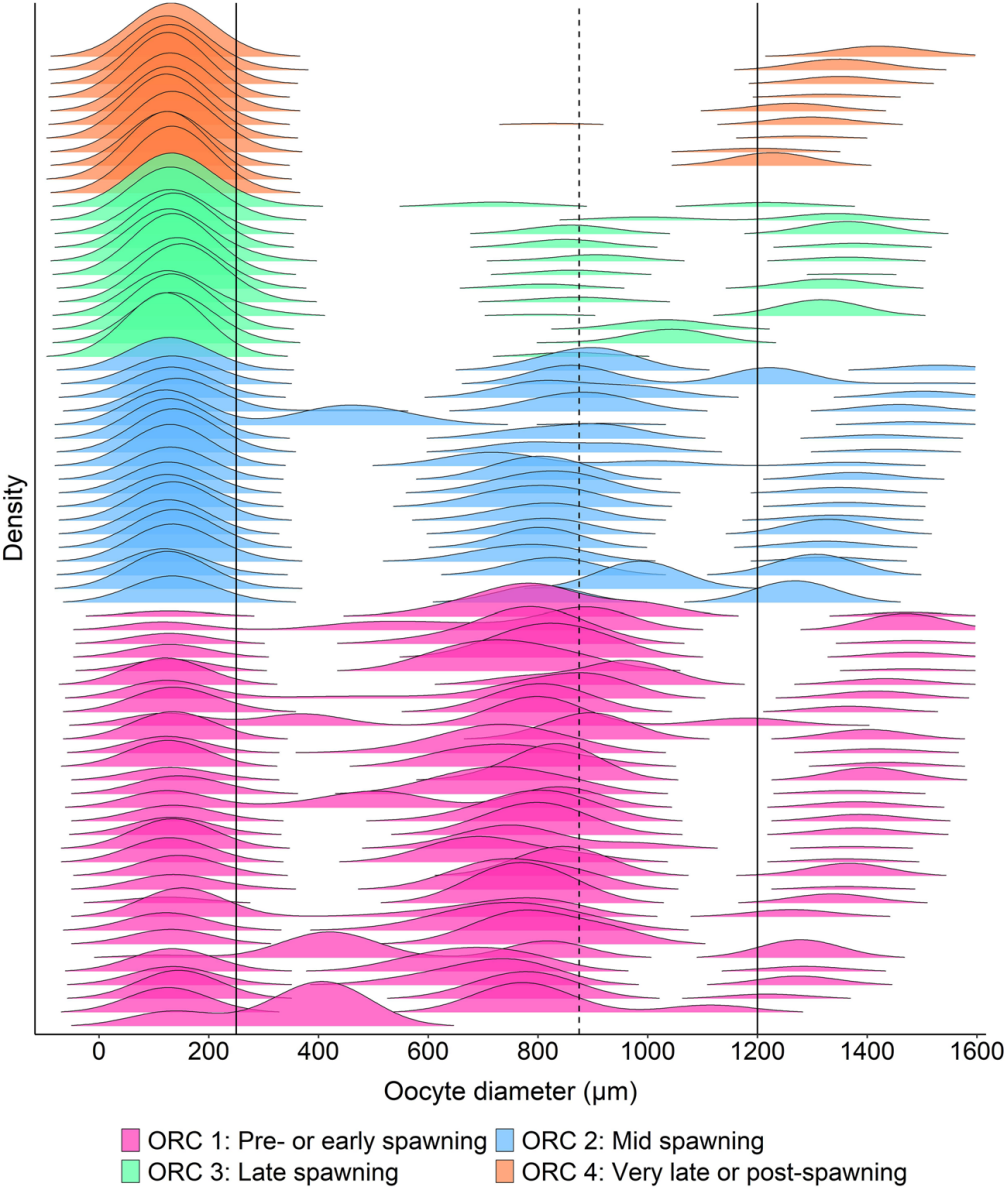
Smoothed OSFD profile plot for all fish sampled in this study. Each OSFD is coloured by ORC, and the order of the plots is based on ascending maximum oocyte diameter within each ORC. Other details as for Fig. 1.

Fish ranked by oocyte ratio category (ORC) had unique OSFD characteristics in several aspects (Table 1, Fig. 2A–I). In ORC 1 (pre-spawning or early-spawning), the proportion of PVOs relative to more mature oocytes in the 250–1200 µm range was lowest, as demonstrated by the presence of relatively large single peak consisting of VOs (Figs. 2A, 3), or a larger proportion of oocytes in the 600–1200 µm range (Figs. 2B,C, 3). As ORC increases, the number of oocytes in the 250–1200 µm range decreases relative to the number of PVOs, and there was a subsequent tendency for a smaller number of oocyte cohorts to be present with increasing ORC (Figs. 2 and 3). In fact, when considering the 500–1200 µm range of oocytes, fish from ORC 3 consistently had a single cohort (Fig. 2F,G), and all ORC 4 fish lacked a distinct bell-shaped cohort (Fig. 2H,I). The pre- and post-spawning fish belonged to ORCs 1 and 4, respectively (Fig. 2A,I), and running fish were partitioned into all four categories (Fig. 2).

**Table 1:** Details for each oocyte ratio category (ORC), including oocyte ratio (OR) range and oocyte size frequency distribution (OSFD) characteristics.

ORC	OR range	Description
1	≤1	*Pre- or early-spawning fish*. Fish with relatively few or no hydrated oocytes will be placed in this category. Between one and three cohorts in the 250–1200 µm oocyte range. Lowest proportion of PVOs to 250–850 µm range oocytes, thus this ORC contains the highest proportion of VOs. It is highly likely that cohorts in the 400–1000 µm range are negatively skewed, i.e. seeing relatively more of those oocytes being larger in size.
2	>1 and ≤3	*Mid-spawning fish*. One or two oocyte cohorts in the 250–1200 µm oocyte range. All fish (with one exception) had an oocyte cohort > 1200 µm. Cohorts in the 400–1000 µm range may be negatively skewed.
3	>3 and ≤15	*Late-spawning fish*. Consistent presence of a single cohort in the 500–1200 µm oocyte range. Store of VOs depleted relative categories 1 and 2.
4	>15	*Very late- or post-spawning fish*. No or very few VOs present relative to PVOs, and any VOs present are not part of a distinct bell-shaped cohort. It is likely that remaining VOs will be reabsorbed rather than spawned. Possible presence of hydrated oocytes.

Representative OSFDs for each ORC are presented in Fig. 2.

Approximately 21% of fish had a VO cohort with a continuous size distribution, but two peaks (*e.g*. Fig. 2C). In all cases, this distribution occurred as a result of oocytes ‘budding off’ when they begin hydration at approximately 875 µm^20^. In some instances, fish had unusual OSFD characteristics that were encountered at low frequency. For example, 4.2% of fish (all in ORC 1) had two VO cohorts that could clearly be separated (*e.g*. Fig. 1C). The term ‘bimodal size distribution’ has been previously used in the literature to describe this characteristic^12^. Furthermore, three ORC 1 fish (4.2%) had an exceptionally long ‘tail’ of oocytes trailing the ~800 µm cohort (*e.g*. Fig. 1D).

### Prevalence and intensity of atresia

**Figure 4:**
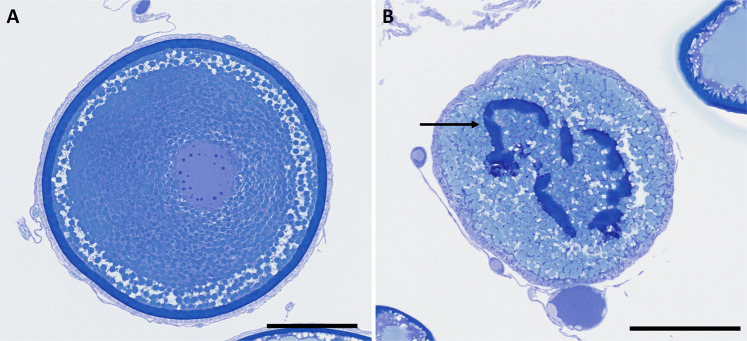
A normal vitellogenic oocyte (**A**) and an atretic oocyte in late alpha residual chorion (LARC) phase (**B**). Arrow indicates chorion remnants. Scale bar = 250 µm.

Atresia was identified in 6 out of 72 ovaries corresponding to a prevalence of 8.3%. In the samples that displayed atretic VOs, reported as mean (SD) intensity (Iα), 5 out of 6 were in ORC 1 (Iα: 3.4 (2.7)%), and one was in ORC 2 (Iα: 5.8%). Overall, Iα ranged from 1.5 to 8.3%. Hence, the lowest and highest Iα values were detected in ORC 1. In comparison with a normal vitellogenic oocyte (Fig. 4A), chorion remnants were observed in the centre of the atretic oocyte (Fig. 4B), or entirely missing in the section plane. These levels of degeneration correspond to the late alpha residual chorion (LARC) and late alpha no chorion (LANC) phases, respectively. Only one ovarian tissue sample (ORC 1) showed early alpha (EA) atresia. None of the 6 fish with atresia had an unusual OSFD pattern.

### Post-ovulatory follicle size and number

**Figure 5:**
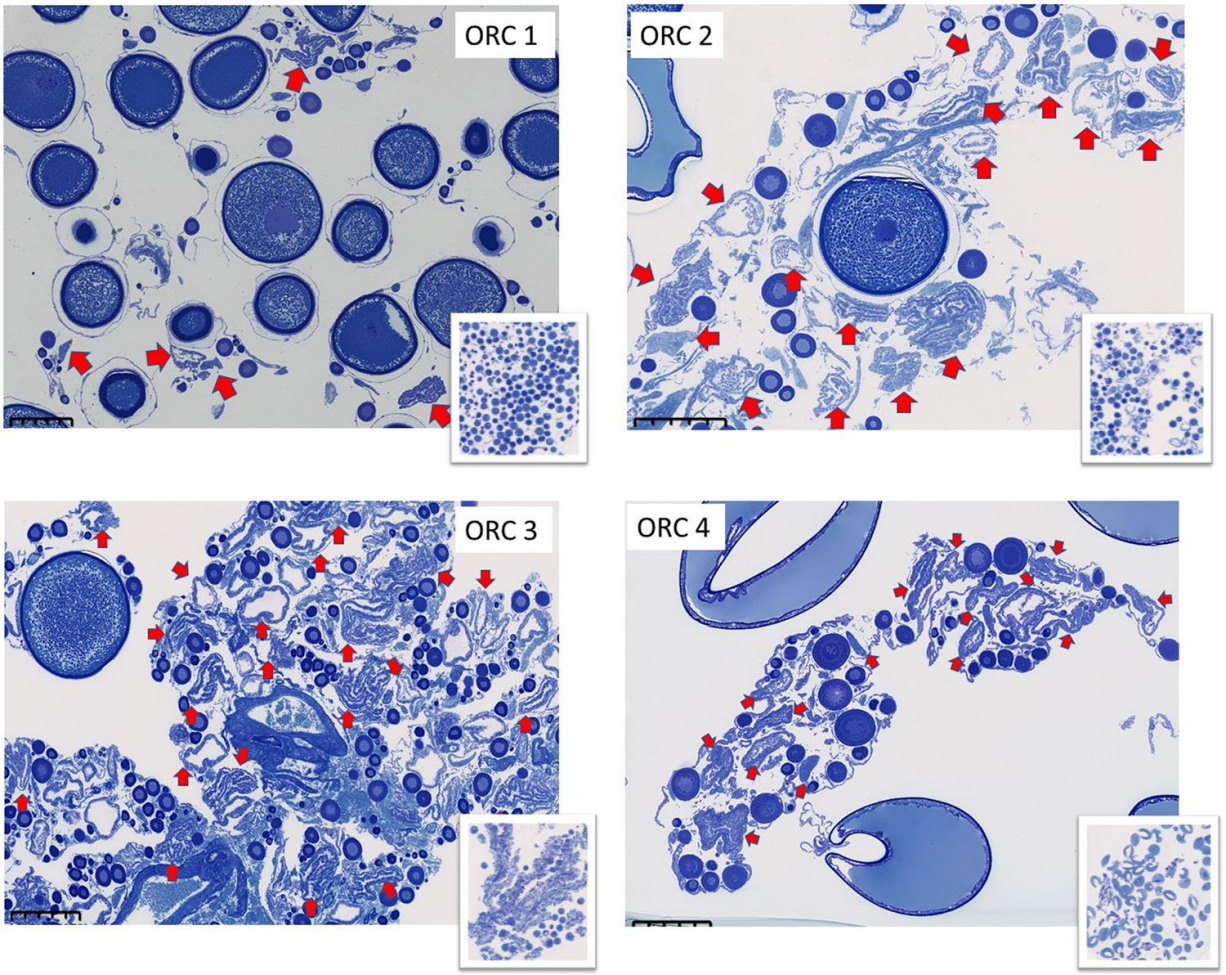
Post-ovulatory follicles (POFs) in each of the four ORCs. Red arrows indicate POFs. Scale bar = 500 µm. Lower magnification micrographs of each representative sample are shown on the bottom right of each panel.

**Figure 6:**
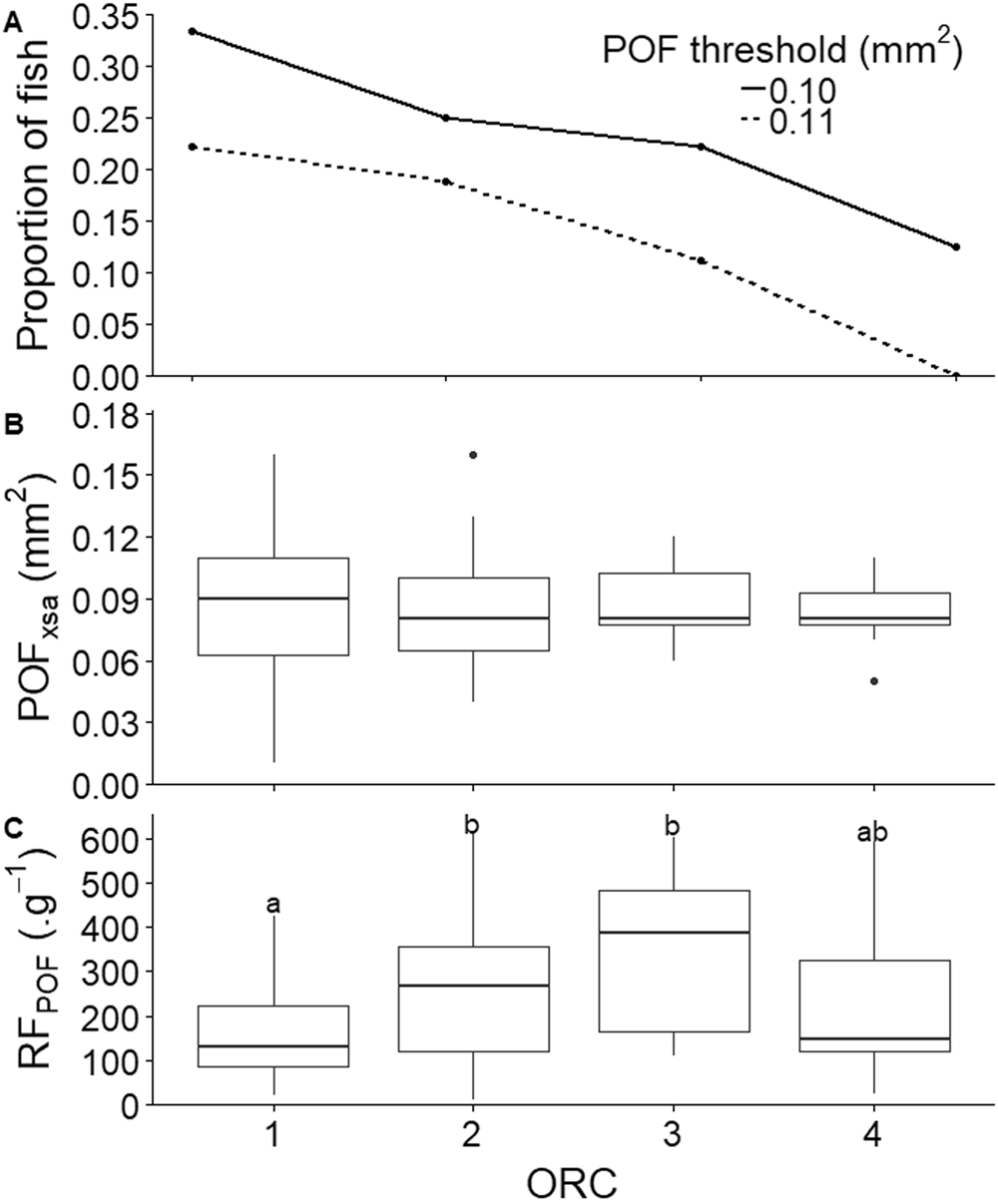
POF metrics (presence, size and fecundity) as a function of ORC. In each ORC: (**A**) The proportion of fish that had a POF larger than a given size threshold (POF_XSA_ > 0.10 or >0.11 mm^2^). This measure can be used as a proxy for the presence of very recent spawners. (**B**) Median post-ovulatory follicle area (POF_XSA_), based on the largest POF from each female, and (**C**) median relative number of post-ovulatory follicles (RF_POF_), when taking all POFs into account. Different superscripts denote statistical significance. Boxplots are in the style of Tukey (median = 50% quantile; upper and lower hinges = 75 and 25% quantile, respectively; whiskers, e.g. upper whisker = largest observation less than or equal to upper hinge + 1.5 × interquartile range).

POFs were detected in the ovaries of fish belonging to all four ORCs (Fig. 5). The fraction of females with new POFs in each ORC decreased with increasing ORC, and the trend was similar for the two POF_XSA_ threshold values used (Fig. 6A). In particular, 34% of females in ORC 1 exhibited POFs larger than 0.10 mm^2^ (and 23% with POFs > 0.11 mm^2^). The decrease in the fraction of large POFs with ORC was almost linear reaching 0.11% for the 0.10 mm^2^ threshold and 0% for the 0.11 mm^2^ threshold, respectively (Fig. 6A). There were no statistically significant differences in size of the largest POF (POF_XSA_) between ORCs (Fig. 6B). However, the number (fecundity) of POFs standardised by whole body size (RF_POF_) showed an inverted-U pattern as a function of ORC, with median RF_POF_ of ORC 1 being statistically different to ORC 2 and 3 (initial p = 0.019; ORC 1 vs ORC 2 p = 0.05; ORC1 vs ORC 3 p = 0.04) (Fig. 6C). Removing the influence of ovarian weight throughout spawning did not change the statistical trend (RF_POF_ vs RF-O_POF_, data not shown). The RF_POF_ vs ORC trend was almost an opposing pattern compared to the proportion of fish with new POFs (Fig. 6A vs C).

### Fish metrics

**Figure 7:**
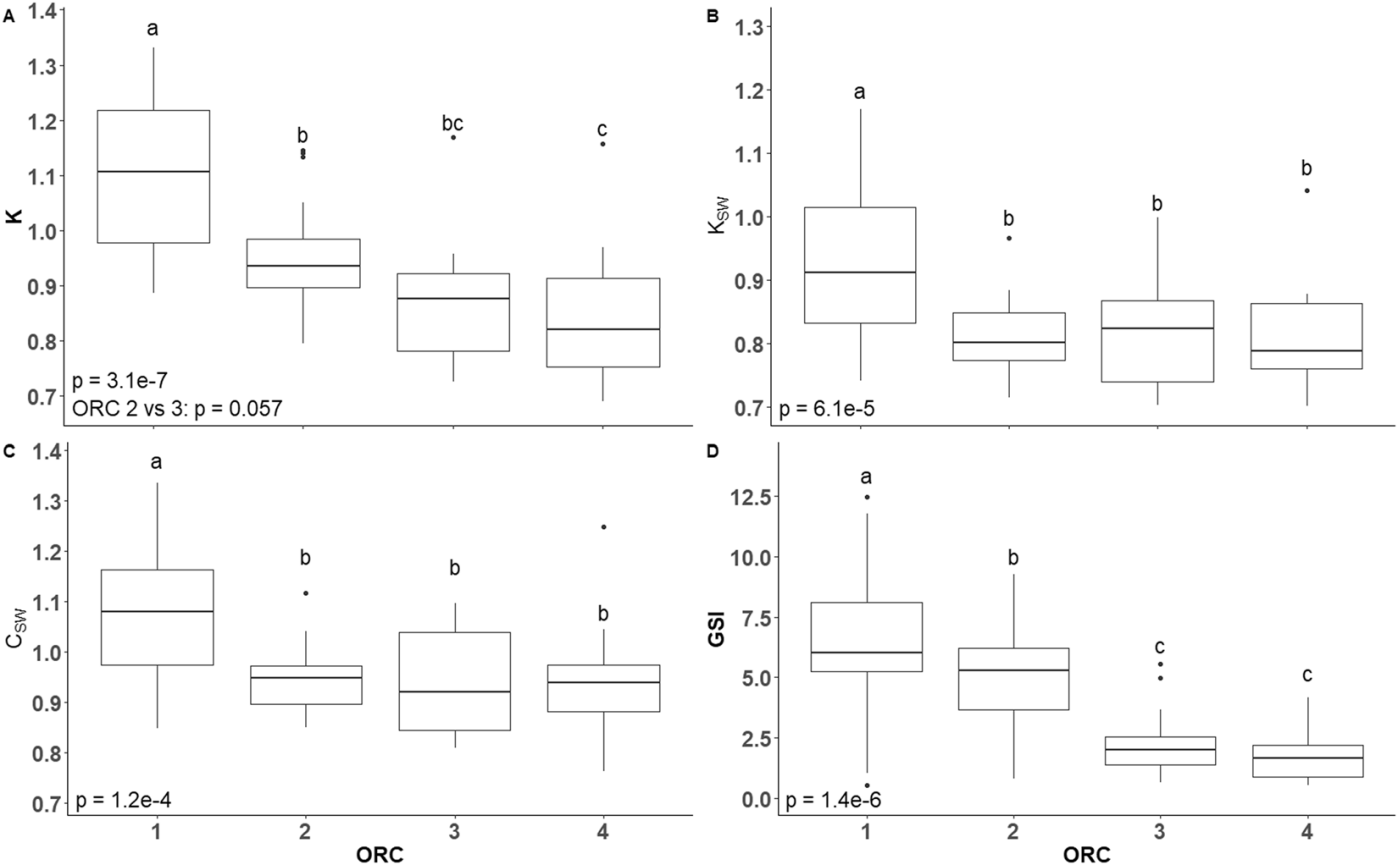
For female fish in each of the four ORCs, (**A**) Fulton’s condition factor (K), (**B**) K after the removal of ovarian influence (K_SW_), (**C**) relative somatic condition (C_SW_), and (**D**) gonadosomatic index (GSI). Different superscripts denote statistical significance, and the initial p-value is indicated on the lower left of each panel.

A comparison of fish metrics between ORCs was performed as a means of validating the ultrametric method, as there was an expectation that GSI and body condition (Fulton’s K) would decrease as spawning progressed and ORC increased. The condition of female fish belonging to ORC 1 was significantly higher than that of fish in ORCs 2–4, regardless of the calculation method used (C_SW_, K or K_SW_, Fig. 7). While the pattern of statistical significance between ORCs was the same for C_SW_ and K_SW_, the pattern for K was different, with an additional pairwise comparison being statistically significant (Fig. 7B,C vs A). Significant differences in GSI were noted between all ORCs except 3 and 4, but the decreasing trend with increasing ORC persisted (Fig. 7D).

## Discussion

The aim of the current study was to develop a novel method of assessing teleost spawning status that is robust to individual variation in oocyte dynamics and can be used at discrete sampling points during the spawning season. In order to do this, 72 *G. morhua* belonging to the Barents Sea cod stock were used as the test species to develop a new ranking system (ORC), that is based on progressive depletion of the VO pool. To understand and validate the new ultrametric classification system, ORC was considered in the context of finer scale ovarian dynamics, *i.e*. OSFD characteristics, including PVOs, and the occurrence of atresia and a spawning marker (POFs). ORC was also related to body condition and GSI, to determine whether these parameters changed as spawning progressed, i.e. ORC increased.

Separation, and subsequent quantification of the PVO fraction in whole mounts is required for the ultrametric method, in contrast to the corresponding quantification of only the VO + FOM fraction which is standard in modern laboratories (see Introduction). By using an optimised ultrasonication protocol, PVOs and larger oocytes were separated from the ovarian connective tissue in a way that did not cause oocyte damage. Ultrasonication has advantages over the use of Gilson’s fluid which has been used for oocyte separation in cod and other species, due to the tendency of Gilson’s fluid to create oocyte ‘tails’ that persist after cleaning, potential loss of atretic and post-ovulatory follicles, incompatibility with histological methods, and long soak time required to break down the connective tissue^8,15,21^. Rigthly so, an ultrasonic cleaning device has already been presented in the literature but was used for VOs rather than PVOs; the protocol in question includes a 40–80 min treatment in tap water^22^. So, the gentle use of the ultrasonic pen therefore opens up for a new era within this field of automated oocyte measurements as PVOs can now be quickly measured and assessed. This is particularly pertinent because evidence is mounting that PVO production is a key factor in understanding fecundity production and style^23,24^. Although our work aimed to properly define spawning status, the present findings also give prospects regarding PVO fecundity estimation (F_PVO_), i.e. number of PVOs in the whole ovary, which currently requires advanced stereology and/or mathematics (packing density theory) to be considered trustworthy^24^. However, refinement of the outlined methodology is likely required to further improve OR accuracy (see issues mentioned in the Methods). In the present context OR, estimated as OR = ∑PVO/∑(VO + FOM), successfully served the purpose to partition into the correct ORC. In pre-spawning fish this expression is reduced to OR = ∑PVO/∑VO. Hence, the corresponding F_PVO_ can then be given as F_PVO_ = OW × OR × OPD_VO_, where OW is whole ovary weight (g) and OPD_VO_ is VO packing density (g^−1^), the latter found from the tight relationship with average VO diameter, using the autodiametric method^12^. Thus, we foresee many spin-offs of the present methodology in years to come.

Following ultrasonication, quantification of the oocytes, and calculation of the OR, female fish were partitioned into one of four ORCs that adequately reflected the properties of their respective OSFDs. For example, as ORC increased and fish approached the end of spawning there was a transition towards a lower number of oocyte cohorts in the 250–1200 µm range which is reflective of VO depletion. The exception to this trend occurred for the only pre-spawning individual caught, which had a single oocyte cohort present in the 250–600 µm range. In this instance, the proportion of PVOs to cortical alveoli (CA) and early VO oocytes was small enough that this fish was correctly partitioned into ORC 1. The ultrametric method can therefore be used to stage fish that are yet to spawn, though in such instances multiple oocyte cohorts >250 µm should not be expected. To use this classification system earlier in the spawning season, further refinement of the OR ranges may be possible to enable differentiation between pre-spawning and early-spawning fish.

Multiple approaches have been adopted in the literature to track the progression of ovarian development over time in both indeterminate and determinate species but were not optimal for assessing reproductive development in this study. For example, when using the hiatus limit method^10^, placement of the hiatus boundaries would have been too subjective for ~15% of fish due to the presence of oocytes between the PVO and larger, more developed VO cohort. In addition, the autodiametric method relies on changes in the mean size, SD and skewness of the VO cohorts over a period of months to track oocyte development prior to oocyte hydration but also during the subsequent act of spawning, i.e. the stage of spawning method^12,15^. In the current study, we observed VO cohorts with a bimodal distribution (two VO cohorts with separate distributions) in just over 4% of the females analysed, and more than 20% of fish had OSFDs where oocytes were budding off/entering FOM, which in turn made the reliable calculation of VO mean, SD and skewness statistics far more complicated (cf. possibly varying threshold values between VOs and FOMs) for a large proportion of fish. While a previous study on *G. morhua* observed bimodal distributions in a few instances just prior to or during spawning, this phenomenon was unexpected and not addressed further at that time^12^. Thus, the ultrametric method circumvents the difficulties associated with the occurrence of ‘low frequency’ OSFD characteristics, i.e. oocyte tails, bimodal oocyte distributions, and the presence of oocytes in the hiatus region. This method can also be applied to ‘running’ fish due to the upper threshold of 1200 µm utilised when calculating OR, which eliminates bias that may be introduced by including free ovum (see Methods). So, although methods undoubtedly exist to reliably assess ovarian progression over a period of months prior to spawning, the present ultrametric method provides a clear advancement in these terms when analysing spawning ovaries, as it overcomes issues associated with traditional methods.

Atresia prevalence observed in the present work (8.3%) is relatively low compared to previous studies (e.g. 28% to 36% in Barents Sea and Baltic cod)^25–27^. Atretic follicles were mostly detected in early-spawning fish (ORC 1) as recently observed in eastern Baltic cod^28^. Fecundity down-regulation before spawning by atresia is a strategy commonly employed in determinate capital spawners^25,28,29^. While atretic oocytes can be found in all stages (PVO, CA or VO^27^), the VO stock is at the highest during early vitellogenesis and then progressively reduced by atresia until stabilisation, close to spawning season^25^. Therefore, it could explain the low atresia prevalence and intensity (1.5–8.3%) observed in the present study during the peak of the spawning season. Moreover, fish with atretic oocytes did not have unusual or ‘low frequency’ OSFDs, such as bimodal vitellogenic cohorts.

POFs are the remnants of the follicular complex after egg release. Given that they apparently have no particular physiological role, POFs are destined to degenerate and finally vanish at a rate that varies with ambient temperature and species^30^. A series of previous studies on various teleosts, including cod, have documented that POF size (POF_XSA_) can serve as an accurate proxy for elapsed time since egg release^17,31^. Even though the process is protracted in species inhabiting cold waters^17,19^, in our study larger POFs were assumed to be younger (i.e. new POFs) and thus closer to a previous spawning event. Concerning the fraction of females with new POFs in each ORC, the trend was quite similar for the two different POF_XSA_ threshold values, with a progressive decrease in the relative production of larger/younger POFs with increasing ORC. This trend in the relative production of younger POFs matched well with the pattern of total POF number (RF_POF_) per ORC. More specifically, RF_POF_ showed an inverted-U pattern as a function of ORC, gradually increasing between ORC 1 and ORC 3 and then decreasing (non-significantly) again in ORC 4. Combining these two sets of results, it appears that the ovaries of ORC 1 fish contain a low total number of POFs which are mostly larger and younger of age. However, females might contain very old POFs from the previous spawning season given that POFs in cod exhibit very low resorption rates^17^. The number of POFs was shown to increase in ORC 2 and ORC 3 whereas the overall production of new POFs was decreasing, apparently suggesting the gradual accumulation of older POFs in cod ovaries during the spawning season. Finally, in ORC 4 the production of new POFs is either minimised (0.10 mm^2^ threshold) or eliminated (0.11 mm^2^ threshold) suggesting that females in this category mainly/only have older POFs. While the reason for the noticed drop in total RF_POF_ from ORC 3 to 4 is unclear, and requires more detailed research insights, it should be noted that this figure contains measurements of both new and older POFs, and other factors such as egg batch size, total number of egg batches, and spawning interval may not have been uniform between fish^32^.

When comparing body condition between fish in the different ORCs, the outcomes of the statistical analyses were dependent on the method of calculation for condition factor. For example, the patterns of significant differences for condition factor between ORCs were the same for the somatic indices C_SW_ and K_SW_, but not K. Specifically, there was a loss of statistical significance in the relationship between condition and TL when the influence of the ovary was removed (K_SW_ vs K), indicating that the dynamic nature of the ovary during spawning significantly influenced K. Furthermore, the use of C_SW_ demonstrated that variation in fish condition was not dependent on TL, and as found by previous works, and the use of C_SW_ eliminated problems that arise due to size dependency in the calculation of K^20,33^.

As *G. morhua* is a capital breeder that uses stored energy for reproduction^18,34^, it was expected that condition would decrease as spawning progressed. In the present study, K decreased as ORC increased, in line with a previous study on Icelandic *G. morhua* that demonstrated the relationship between increasing PVO proportion and decreasing K^10^. Without the influence of the gonad, C_SW_ and K_SW_ were both highest in the pre and early-spawning group (ORC 1), suggesting that significant somatic energetic resources were used during the earlier stages of spawning. In *G. morhua*, the rate and magnitude of change in condition factor throughout spawning depends on several factors, including starting condition, size, age, and environmental factors^35–37^, though the reduction in condition was consistent for fish sampled in this study. Similarly, GSI decreased markedly as ORC increased, which is reflective of serial egg release over the spawning season, a well-documented phenomenon in cod^10,19^.

Using our newly developed method, female fish were successfully partitioned into one of four ORCs that accurately reflected the characteristics of their OSFDs and spawning stage. The ultrametric method overcomes difficulties associated with presence of bimodal oocyte distributions, oocyte tails, lack of clear hiatus region, and presence of free ova, and can be implemented at a single discreet sampling point. A significant proportion of the workflow (oocyte counting) is also fully automated, and the technique may circumvent the need for histological analysis depending on the desired outcome. For these reasons, the ultrametric method is a cost-effective and rapid tool that can be used to assess preserved ovarian samples. Notably, we detected examples of cod females that apparently showed signs of *de novo* oocyte recruitment or had bimodal VO cohorts, questioning that cod is a strict determinate spawner. In this work we do not specifically address the drivers behind these unusual patterns, though there are multiple possibilities, e.g. a warmer climate or improved body condition. We believe that the present method advancements could be successfully used in new projects to address such research questions, and related ones. Furthermore, while reproductive dynamics are known to vary across stocks and species, the current principle of relating advancements in spawning (depletion of VOs and FOMs: denominator) to the standing stock of PVOs (numerator) should be broadly applicable to determinate species other than cod, and possibly to indeterminate spawners, as long as the fraction of PVOs entering vitellogenesis during spawning^24^ is assessed by other means first. Thus, we expect that the ultrametric method will be compatible with a wide range of species, though the OR thresholds should be adjusted as needed to ensure that species-specific differences in ovarian dynamics are accounted for. Fine adjustment may also be required when applying this method to fish from different stocks or environmental conditions.

## Methods

### Sample collection

This work targeted Northeast Arctic (Barents Sea) cod, based on following characterisation of otolith zonation^38^, migrating to the coast of northern Norway to spawn during winter time^39^. Seventy-two females of this stock type were collected from the southern Lofoten archipelago (Norway, Røst municipality) by commercial fishing vessels, using gill nets, during the peak spawning season (6^th^ and 7^th^ April 2018) as part of the Institute of Marine Research’s coastal sampling program of groundfish landings run by the chartered vessel ‘*Falkungen’*. Capture site coordinates were: 67°23′04.0′′N and 12°02′04.0′′E, 67°32′00.0′′N and 11°54′00.0′′E, 67°29′05.0′′N and 11°43′04.0′′E, and 67°25′07.0′′N and 12°08′08.0′′E. Most females were 9 + (7–16) years and around 96 (63–126) cm in total length. For each individual, a section of ovarian tissue was taken from the mid-section of the right lobe and stored in 3.6% neutral buffered formaldehyde (the tissue/formalin ratio being 1:10) for later analysis. For fish that were not ‘running’, that is, their gonads did not contain free hydrated (ovulated) oocytes, a single tissue sample (≈1–2 g) was deemed suitable as ovarian homogeneity has been previously demonstrated for *G. morhua*^40^. For running fish, an additional sample (≈1 cm^3^) was taken using a pipette and stored in the same way as dissected tissue samples. As the fish utilised by this study were captured as part of a traditional fishery, fishing rights are attributed to each commercial vessel. In addition, all animals were deceased at landing in accordance with standard fishing practices. As such, animal ethics approval for this research initiative was not required.

### Ultrasonication and autodiametric analysis of ovarian samples

Oocytes are bound to connective tissue which makes them difficult to count and measure. Thus, ultrasonication was tested as a means of oocyte separation on 5 samples, and the impact of ultrasonication was assessed by measuring oocyte integrity and comparing their OSDFs before and after treatment. Firstly, a subsample of oocytes in formalin-fixed tissue were separated using an ultrasonic pen (Vibra-Cell VCX 130FSJ, Sonics & Materials Inc., US: 130 Watt, 50% amplitude) for 10 s. Then 40 µl of toluidine blue was added to ensure that PVOs would also be detectable. Samples were then kept in 74 µm-mesh netwells (Corning Inc., USA) while they underwent a series of formaldehyde washes to remove excess stain. This implied that PVOs < ≈75 µm should be considered lost, and the lower threshold value was conservatively set at 100 µm for oocyte counting. Following ultrasonication and staining, whole-mount OSFDs were generated as described previously^12^, with some modifications. Three micrographs were taken for each sample so that a minimum of 200 oocytes were photographed per fish (Leica stereomicroscope equipped with a digital camera system). Oocyte diameters (OD) were automatically measured using the open source image analysis program ImageJ (v. 1.52, https://imagej.nih.gov/ij/) with the plugin ObjectJ (https://sils.fnwi.uva.nl/bcb/objectj/) and an adapted variant of the elliptical oocytes project (https://sils.fnwi.uva.nl/bcb/objectj/examples/oocytes/Oocytes.htm). Pictures had a resolution of 0.1803 px/µm and oocytes were selected based on threshold values for size (100–1600 µm), grey tone (<111), roundness (0.8–1.0) and ellipticity (≥0.91).

**Figure 8:**
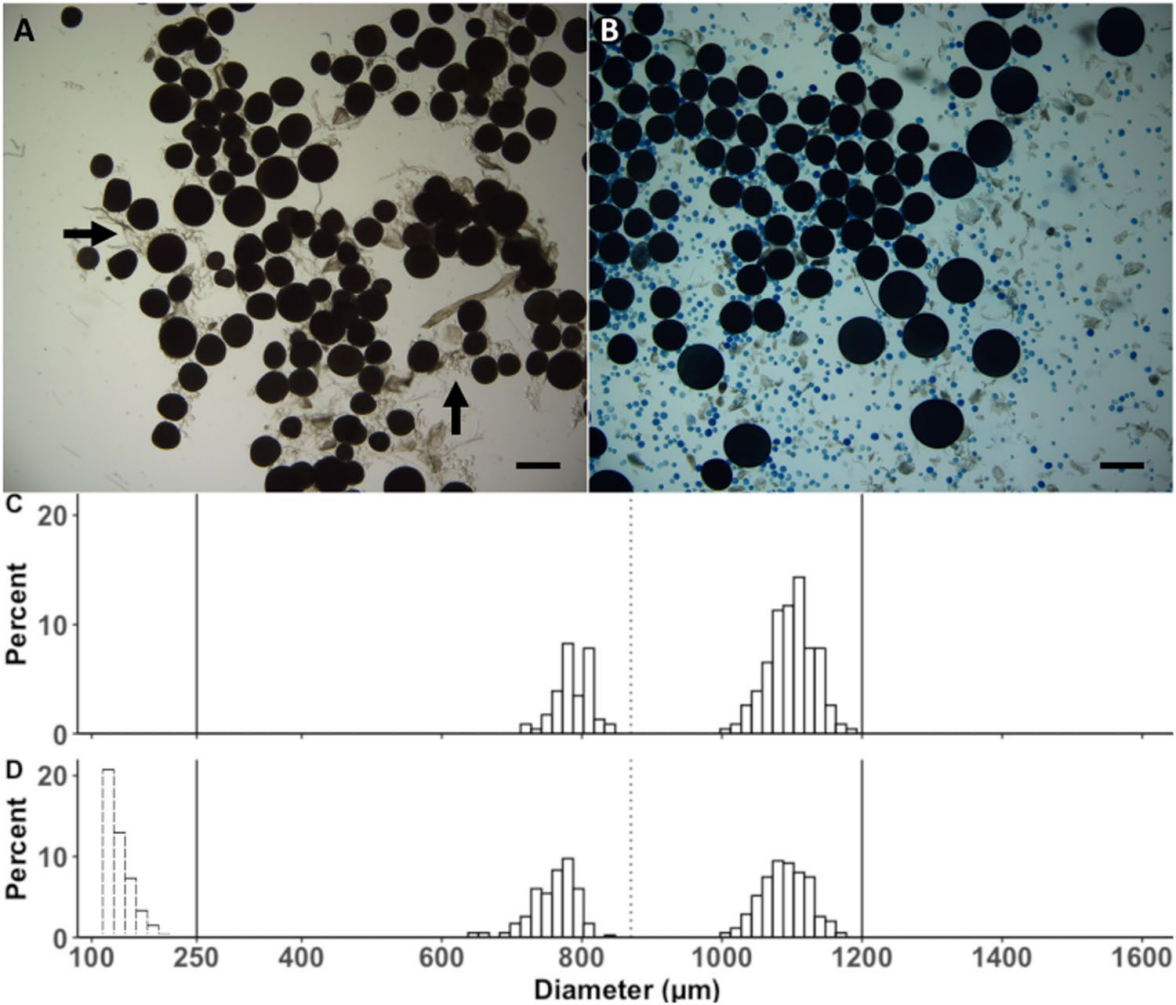
The effect of ultrasonication on oocyte integrity and countability. Representative whole-mount micrographs of ovarian samples before (**A**) and after (**B**) ultrasonication treatment and staining. (**A**) Small and transparent PVOs are densely packed and hardly noticeable (black arrow). (**B**) After ultrasonication and staining, PVOs are well defined and separated. Scale bar = 1000 µm. Oocyte size frequency distribution of a representative fish before (**C**) and after (**D**) ultrasonication and staining. In D the distribution of PVOs following ultrasonication is indicated by the dashed bars. Other details as for Fig. 1.

**Figure 9:**
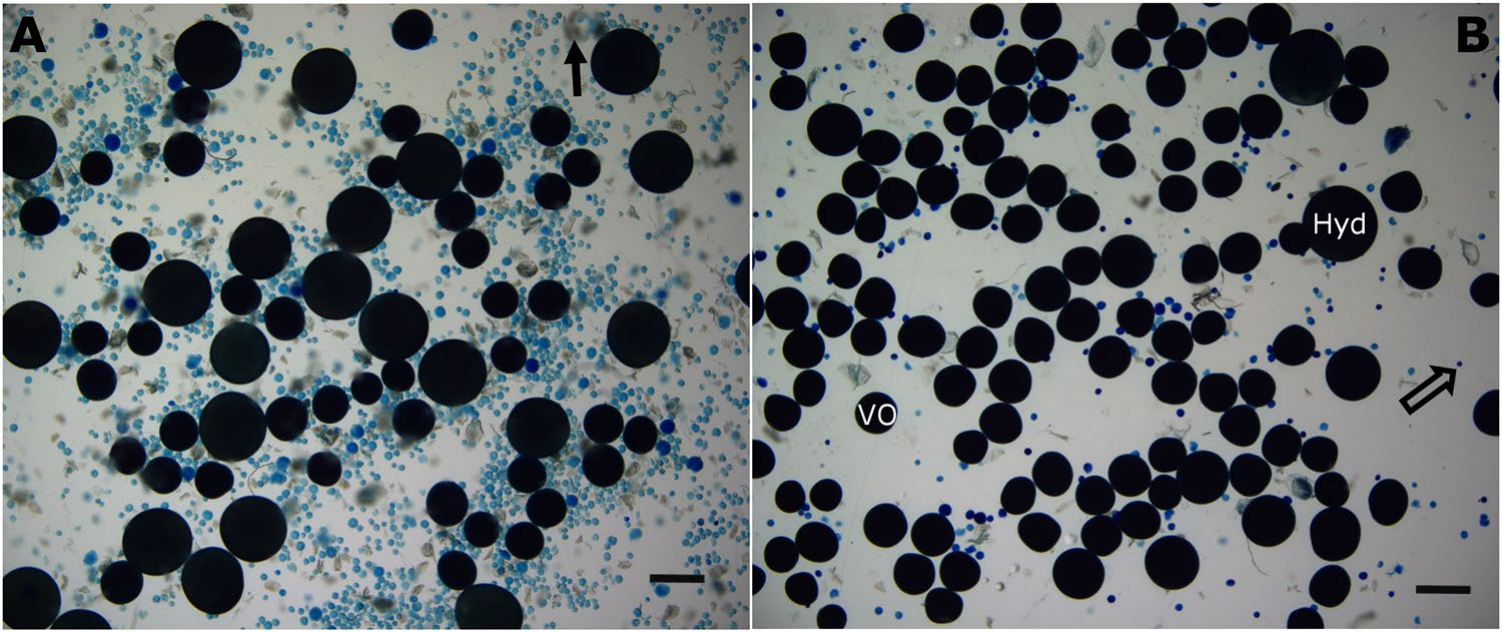
Representative whole-mount micrographs of stained samples with a relatively high **(A**) and low (**B**) previtellogenic oocyte (PVO) count. PVO (hollow arrow), vitellogenic (VO), and hydrated (HYD) oocytes are indicated (in **B**), as is ovarian debris (solid arrow) (in **A**). Scale bar = 1000 µm.

Micrograph analysis revealed that ten seconds of ultrasonication could in cases cause the unwanted formation of small cracks in few of the advanced VOs and oocytes undergoing final oocyte maturation (FOM), whereas PVOs appeared to be more robust to this treatment. To avoid any oocyte damage, the length of ultrasonication was reduced to 5 s when advanced VOs or FOMs were present, and the oocyte integrity and composition was re-examined as above. Following these adjustments, no change in oocyte integrity was observed as attested statistically by the absence of difference in the leading cohort (LC) OD (Wilcoxon test for repeated measurements, p-value = 0.063) (Fig. 8). However, as expected, there was a statistically significant change in overall OSFD before and after ultrasonic pen treatment (Wilcoxon test for repeated measurements, p-value = 2.6e^−8^) due to an increase in the countability of smaller oocytes (<800 µm) following ultrasonication and staining (Fig. 8). All remaining samples were processed using the optimised procedure. In instances where debris (such as connective tissue) was measured, the micrograph was manually cleaned. For samples that contained a very high proportion of PVOs, counting efficiency was reduced to some extent for the PVO fraction due to oocyte crowding (Fig. 9).

### Development of a novel method for assessing stage of spawning

We developed a new measure, termed oocyte ratio (OR), which was calculated by dividing the total number of oocytes present in the <250 µm fraction (PVOs) by the total number of oocytes present in the more mature 250–1200 µm fraction. All counts were determined using advanced image analysis systems on single ovarian tissue sub-samples (see above): OR = ∑PVO/∑(VO + FOM). As *G. morhua* is traditionally considered to be a determinate species with plentiful PVOs^41^, we theorised that the total number of PVOs will remain relatively stable, whereas the total number of more advanced oocytes should decrease as batches of eggs are released over the spawning period. As a result, the OR should increase as fish approach the end of spawning, and this measure can be used to rank/classify fish and assess spawning dynamics. For OR calculation, the upper OD limit of 1200 µm was chosen to exclude free hydrated eggs that were sampled using a pipette, as the proportion of this fraction to sectioned tissue may have varied between samples and would complicate the analysis (similar to what has been previously noted when using dissected tissue to assess fecundity in *G. morhua*^42^). For the lower threshold, 250 µm was selected as this fraction contains only primary growth oocytes, i.e. has been previously used to differentiate between immature and developing *G. morhua*^41^. It should be noted that a lower threshold of 200 µm was also tested, but the higher threshold allowed a clearer separation between fish of different developmental stage. One fish had no oocytes in the 250–1200 µm range, and in this instance the value of zero was changed to 1 so that an OR could be calculated.

In order to gain further insight into fecundity formation patterns, the OR was used to rank female fish and subsequently split them into four oocyte ratio categories (ORCs) that are defined by features of their OSFDs (Table 1). As previously mentioned, PVO counting efficiency was reduced to some extent for some samples that contained debris or had a very high proportion of PVOs. However, due to the dramatic difference in the number of PVOs between fish from different ORCs (see representative micrographs in Fig. 9), we can be confident in the OR rank given to each fish, and subsequent partitioning of fish into the four ORCs.

### Histological analysis of ovarian tissue

A histological approach was utilised to generate accurate data regarding oocyte stage^6^ and provide information about ovary dynamics including fecundity down-regulation by atresia^8^ and the presence of POFs^17^. This analysis enabled crosschecking with the whole-mount OSFD data produced in the previous steps. As such, fixed ovarian tissue was processed according to a previously published protocol^41^. Briefly, subsamples were progressively dehydrated in a series of ascending alcohol, infiltrated and embedded in 2-hydroxyethyl methacrylate (Technovit 7100, Heraeus Kulzer GmbH, Germany). Sections (4 μm) were then obtained using a microtome Leica RM 2255 and stained with 2% toluidine blue and 1% sodium tetraborate. Finally, slides were scanned using a slide scanner (Hamamatsu S60) with a x40 objective and a resolution of 220 nm/pixel.

Oocyte measurements were performed on the 5 largest oocytes that had a clearly visible nucleus, cf. ‘leading cohort’^12^ (see above). The prevalence of vitellogenic atresia (‘atresia’) was defined as the proportion of females with α atretic oocytes, sub-grouped into early alpha (EA), late alpha residual chorion (LARC) and late alpha no chorion (LANC)^43^, in relation to the total number of females studied^44^. The intensity of α atresia (Iα) was thereafter calculated as Iα = N_i_/(N_i_ + N_j_), where N_i_ is number of α atretic oocytes and N_j_ is the number of normal oocytes^8^. The NDP.view2 Viewing software was utilised for all image analyses (Hamamatsu).

POFs were measured and quantified. The number of POFs (POF fecundity; F_POF_) was determined stereologically by the Weibel method^45^ using a grid of 256 points and 10 counting fields of 6 mm^2^ each^46^. In the estimation of ovary volume (see formulae in the references provided^40^), ovary specific gravity was set at 1.0464 g cm^−3^. POF size was calculated as the cross-sectional area of the largest POF from each fish (reported as median POF_XSA_ for each ORC), i.e. assuming in these cases an equatorial transection. The percentage of fish that had a POF larger than a given size threshold (POF_XSA_ > 0.10 and >0.11 mm^2^) was also plotted for each ORC, which was used to assess the prevalence of very recent spawners. Relative POF fecundity (RF_POF_) was defined as RF_POF_ = F_POF_/BW, where BW is whole body weight (in g). As total BW is influenced by changes in ovarian weight throughout spawning, RF-O_POF_ was also calculated as RF-O_POF_ = F_POF_/(BW-OW). Unlike POF_XSA_, these measures take into account all POFs present.

### Calculation of fish metrics

The BW ‒ total length (TL) relationship was established by applying a linear regression to a scatter plot of log-transformed data. The exponent (slope) for the equation (R^2^ = 0.88, P < 0.001) was 3.42 and the 95% confidence interval (CI) of the exponent was 0.30. Hence, the lower limit of the CI was 3.12. As such, using an exponent of 3 to calculate Fulton’s K (K) (see below) would be an inaccurate approach for calculating fish condition. Therefore, we used an approach similar to that of Kjesbu *et al*. (1998)^42^, and subtracted ovary weight (OW) from BW, to remove the substantial influence of the ovary during the spawning period. Thus, the expected somatic weight (SW) of female fish can be calculated using cube law: *SW*_*expected*_ = *aTL*^*b*^, where ‘a’ (e^−5.82) is the constant (regression intercept), and ‘b’ (3.23) is the slope. The 95% CI for the exponent was 0.22, and the R^2^ was 0.91. Relative somatic fish condition (C_SW_) was then calculated using published methods^33,47^ except that SW was used instead of BW: *C*_*SW*_ = *SW*_*observed*_*/SW*_*expected*_. When using this method of calculation, observed and expected weights are equal when C_SW_ is 1, a number smaller than 1 implies lower than expected condition, while the inverse is true for values greater than 1. As K is a familiar and commonly used measure of fish condition, results from statistical tests that utilised C_SW_, K and K without the influence of the ovary (K_SW_) were compared. GSI was calculated as: *GSI* = *(OW/TL*^*3.23*^*) x 10*^4^. The more resilient measure of TL was used in the place of BW^48^, and a scaling constant was incorporated^49^.

### Graphing and data analysis

All statistical analyses and figure production were carried out in R v3.4.4/RStudio v1.1.447^50^. Figures were created using a combination of the R packages ggplot2^51^, ggridges^52^, cowplot^53^, and ggthemes^54^. Differences in POF data and fish metrics between ORCs were assessed non-parametrically using the Kruskal-Wallis test, and if the result was significant a Pairwise Wilcoxon Rank Sum Test was performed in conjunction with the Benjamini-Hochberg post-hoc adjustment to reduce the risk of Type 1 error.
